# Roles of Mutation, Ploidy, and Recombination in Adaptive Evolution in Two Divergent Model Yeasts

**DOI:** 10.3390/genes17020204

**Published:** 2026-02-08

**Authors:** Megan Hitchcock, Jianping Xu

**Affiliations:** Department of Biology, McMaster University, Hamilton, ON L8S 4K1, Canada; hitchcm@mcmaster.ca

**Keywords:** Baker’s yeast, cryptococcosis, mutation accumulation, whole-genome sequencing, mitotic recombination, sexual reproduction, haploidy, diploidy

## Abstract

Genetic variation underlies the capacity of populations to adapt, yet what drives how this variation is generated and maintained in natural populations remains poorly understood. Fundamental processes such as mutation, ploidy, and recombination are known to shape genetic variation and adaptive potential but are typically studied in isolation and under controlled laboratory conditions. How these processes act together under varying environmental conditions to structure genetic variation across complex natural populations remains unresolved. In yeasts, these processes are dependent on reproductive mode, ploidy shifts, and environmental stressors, which jointly shape genomic stability and adaptive potential. Here, we review our current knowledge on the roles of mutation, ploidy, and recombination in adaptation in the model yeasts *Saccharomyces cerevisiae* and the human pathogenic *Cryptococcus*. We highlight heterogeneity in mutation rates, recombination, and ploidy states across strains, environments, and populations, challenging the assumption that these parameters are uniform. We argue that fluctuating environments, increasingly driven by climate change, are likely to intensify interactions among these processes to impact evolution in ways that remain difficult to predict. Integrating population genomics with ecologically realistic frameworks will be essential for understanding natural evolutionary dynamics and anticipating fungal adaptation and disease emergence.

## 1. Introduction

Evolution is the change in genetic material over time and is a fundamental component of biology. Even before heredity was understood at the molecular level, Darwin introduced life as a dynamic system shaped by descent with modification, fundamentally altering how biological diversity was viewed [1]. Yet, it still took until the mid-20^th^ century for Avery, MacLeod, and McCarty to demonstrate that DNA is the hereditary material underlying biological variation, which allows evolution to occur [2]. As genetics and molecular biology continue to advance, our appreciation for the complexity of this system has only increased. In 1968, Kimura, followed by King and Jukes, independently proposed the Neutral Theory of Evolution, which argues that most genetic changes are selectively neutral [3,4]. This theory highlights spontaneous mutation and genetic drift as playing central roles in evolution, shifting the framework away from selection as the dominant force. However, though stochastic processes are increasingly recognized as key to understanding natural genetic variations, adaptation driven by selection continues to be a fundamental aspect of evolutionary change [5]. Adaptation is the process where a population increases its fitness relative to others in the immediate environment by acting on genetic variations among individuals [5]. Thus, a population’s capacity for adaptation is directly related to the available genetic variation present in the population. This variation is shaped by many biological processes, including mutation, recombination and ploidy level.

Yeasts are unicellular fungi. They are phylogenetically diverse and have been found to inhabit a wide range of ecological niches, including those associated with human activities [6,7,8]. For example, domesticated nearly 9000 years ago for alcoholic fermentation, the budding yeast *Saccharomyces cerevisiae* has since become central to baking, brewing, cheese making, and many other applications [9,10,11,12]. However, the same adaptive potential also contributes to the evolution of pathogenicity, which establishes many yeast species as human pathogens [13,14,15]. Although uncommon, isolates of *S. cerevisiae* are also observed in clinics [16,17]. Invasive fungal infections caused by yeasts are estimated to contribute to more than one million deaths worldwide annually [18]. Among these, the human pathogenic *Cryptococcus* (HPC), which comprises seven species, can cause cryptococcal meningitis, a leading cause of mortality among HIV/AIDS+ individuals [19,20]. More recently, the emergence of the multidrug-resistant yeast *Candidozyma auris* (syn. *Candida auris*), a species first reported in 2009, and its global spread illustrate how yeasts can evolve into critical public health threats [21]. Climate change is expected to amplify these evolutionary pressures by shifting environmental conditions and altering selective pressures, such as temperature and humidity [22]. These shifts are predicted to influence both pathogenic and domesticated species, underscoring the need to understand how yeast populations respond to stress to predict future disease risk and antifungal resistance.

Increased genetic variation in natural pathogen populations can increase adaptive potential, enabling rapid evolutionary responses to host immune defences and antifungal use. Elevated genetic variation increases the likelihood that drug-resistant or ecologically adapted genotypes are present prior to infection–treatment intervention, allowing pathogens to quickly respond to treatments or the host immune response [23,24]. This genetic heterogeneity complicates treatment strategies and pathogen surveillance, while cryptic and unsampled variation in natural reservoirs reduces the predictability of pathogen evolution. As a result, public health responses, which must defend against ongoing and pre-existing adaptive mutations, can lag pathogen adaptation. Thus, increased natural genetic variation creates a challenge for the management of pathogenic yeast populations. Yet, how genetic variation is increased and maintained across natural reservoirs remain largely unresolved. Additionally, understanding how genetic variation is generated and contributes to adaptation in response to stress is fundamental for our understanding of evolution. Mutation, ploidy shifts and recombination are not independent processes but rather interact with each other to influence genetic variation. The divergent yeasts *S. cerevisiae* and HPC represent model yeasts from which to investigate the combined impact of these processes on evolution.

Both *S. cerevisiae* and HPC have well-annotated genomes, robust laboratory strains and are recognized as model systems used to study molecular genetics. Each yeast can reproduce both asexually and sexually, are observed to undergo ploidy variation, and contain pathogenic and non-pathogenic strains. In addition, *S. cerevisiae* and HPC represent the two largest divergent fungal phyla Ascomycota and Basidiomycota, respectively. They differ in their natural ploidy states and are associated with distinct ecological and anthropological conditions, subjecting them to different selective pressures. These shared and divergent traits make *S. cerevisiae* and HPC strong candidates to compare conserved evolutionary processes while contrasting between key parameters such as natural ploidy state and environmental pressures. Laboratory experiments of *S. cerevisiae* are central for understanding fundamental principles of yeast genetics, while HPC provides a system for evaluating these principles in the context of human pathogens.

In this review, we first introduce the life cycles of *S. cerevisiae* and HPC. This is followed by reviewing and discussing the process of mutation, the influence of ploidy levels, and the role of recombination in shaping the adaptive potentials of these organisms. Understanding these processes and their mechanisms is critical, as we anticipate an increased rate of evolution under changing environmental conditions.

## 2. Life Cycle

**Figure 1:**
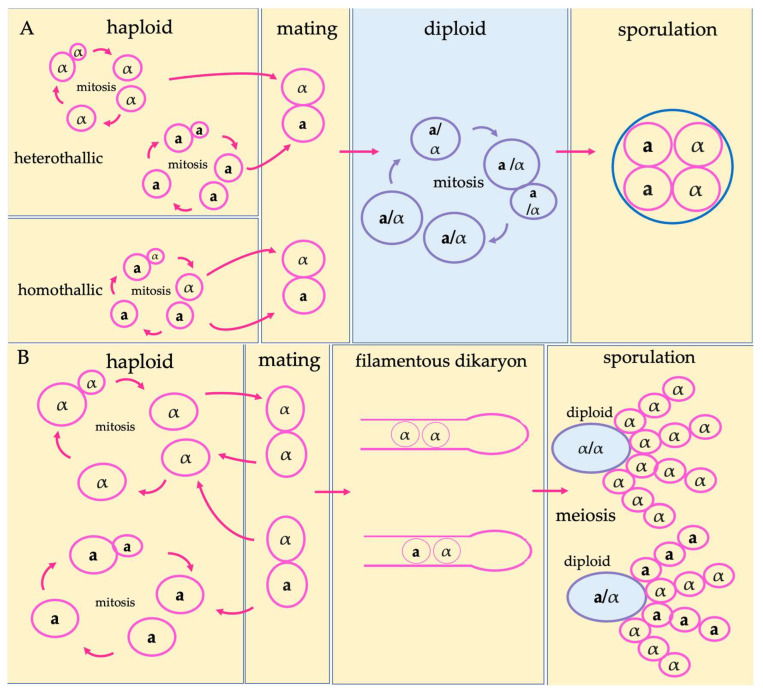
Schematic life cycles of the two model yeasts reviewed in this study. (**A**) The life cycle of *Saccharomyces cerevisiae*, including both heterothallic and homothallic cycles. (**B**) The life cycle of the human pathogenic *Cryptococcus*, representing both a–α and α–α mating.

To help understand the three processes to be reviewed in this study, we first outline the life cycles of *S. cerevisiae* and HPC, as these cycles provide the framework for describing when mutations arise and how recombination and ploidy shifts occur. Figure 1 provides a visualize summary of this section. Both yeasts possess two mating types, *MAT***a** and *MAT*α, specified at the mating-type locus (*MAT*) [25,26].

The life cycle of *S. cerevisiae* involves regular alternation between haploid and diploid states, with the diploid state considered the more stable one of the two [26]. In nutrient-rich conditions, both haploid and diploid cells can proliferate mitotically by budding. Under nutrient limitation, diploid mitotic growth will arrest and transition to meiosis, producing haploid ascospores. When conducive conditions arise, mating between *MAT*α and *MAT***a** haploid cells will occur, fusing to restore diploidy [26]. Both heterothallic and homothallic strains of *S. cerevisiae* occur in nature. Heterothallic strains produce daughter cells that retain the same mating type, whereas homothallic strains can spontaneously switch mating type followed by a double-strand break at the *MAT* locus that is repaired by gene conversion with a mating-type donor locus. The potential for homothallic strains to switch mating types enables mating and self-fertilization between recently divided sister cells containing opposite mating types [26].

In contrast, HPC cells are predominantly haploid, with a heterothallic mating system, meaning that there is no mating-type switching [25]. The canonical sexual cycle involves fusion between *MAT***a** and *MAT*α cells, followed by the formation of filamentous dikaryotic hyphae and brief diploid formation within the basidium, which quickly undergo meiosis to produce haploid basidiospores [27]. However, accumulating evidence indicates that (α–α) unisexual reproduction also occurs in nature [25]. Initiation of the sexual cycle is generally associated with stressful conditions. Diploid cells of *Cryptococcus* spp. are rare and often associated with hybridization between divergent lineages where sequence divergence results in disrupted meiosis [25].

For both yeasts, most reproduction is thought to be clonal, through asexual budding or selfing, with outcrossing being rare [26,28]. However, the relative rates of clonal versus sexual reproduction in natural populations remain largely unknown. This mixture of reproductive modes and shift in ploidy creates a complex evolutionary system where sexual reproduction can generate genetically diverse progeny for selection to act, while clonal proliferation then amplifies genotypes with advantageous mutations or allelic combinations. The occasional return to sexual reproduction then allows for recombining genetic materials, which can facilitate adaptation and reduce the accumulation of deleterious mutations [29,30]. Taken together, the reproductive strategies of *S. cerevisiae* and HPC shape the timing and frequency of genetic change. Because the life cycle is thought to be dependent on environmental conditions and changing conditions are predicted to increase the ecological range of lineage boundaries, increasing the likelihood of divergent strains interacting, climate change may alter natural rates of sexual reproduction, further contributing to increased genetic variation. To understand how these differences translate into evolutionary potential, the following sections explore the three key processes that generate genetic variation: mutation, ploidy shifts, and recombination.

## 3. Mutation

Mutations are the ultimate source of genetic variation that enable evolutionary change. Although most mutations are neutral or deleterious, rare beneficial mutations are produced and are necessary for adaptation. Mutations include both structural changes in the genome, such as deletions, insertions, inversions, translocations, and duplications, as well as single-nucleotide changes [31]. Mutations arise spontaneously throughout the cell cycle during DNA replication and repair, and by exogenous factors, such as exposure to environmental mutagens, which can significantly elevate the mutation rate [31].

The mutation rate can be measured using different approaches and the rate can vary depending on the analyzed strains, traits, and environmental conditions. A common measure of the mutation rate refers to the number of mutations per site per generation and reflects how frequently genetic changes arise. It is a key parameter in evolutionary biology because it directly influences the rate at which populations accumulate genetic variation [32]. An organism’s baseline mutation rate is a balance between endogenous DNA damage and the fidelity of DNA replication and repair machinery. Due to the chemical instability of DNA, endogenous damage can occur by hydrolysis, depurination, deamination and reactive oxygen species [33]. To offset this damage, many DNA repair pathways are implemented to maintain genomic integrity, including mismatch repair, base excision repair, nucleotide excision repair, homologous recombination, and non-homologous end joining systems [34,35]. Mutations increase when replication errors evade proofreading or when repair is incomplete or error-prone. Thus, the mutation rate is the net outcome of these systems and any modifications to these pathways can alter the mutation rate [36].

Mutations can also arise through stress-induced mutagenesis, in which exogenous stressors disrupt repair systems or directly damage DNA. Such stressors can include proteotoxic and oxidative stress, chemical agents, temperature shock, and exposure to antifungal drugs [37,38,39,40]. Exogenous stressors can act by directly damaging DNA and increasing the reliance on repair pathways, or they can influence the function of proteins associated with replication and repair fidelity [41]. For example, high-temperature shock can disrupt protein folding and the enzymatic activity associated with fidelity [42]. How stressors are expected to impact the mutation rate depends on if they directly damage DNA, influence replication and repair proteins, or do both.

Mutations in genes, which lead to increasing endogenous DNA damage or compromise the fidelity of DNA replication and repair, can lead to elevated mutation rates and are typically referred to as mutator alleles. These alleles can act either by increasing the burden of DNA that require repair or by reducing the fidelity of replication and repair processes. Mutator alleles are often considered detrimental because the increased accumulation of deleterious mutations can substantially reduce organismal fitness [43]. However, in large populations or rapidly changing environments, it is hypothesized that mutator alleles may confer a selective advantage by increasing the rate of novel genetic variation, some of which may be beneficial for selection to act [43,44]. Furthermore, strains with significantly increased mutation rates are referred to as hypermutator strains.

Mutation rates are commonly estimated using laboratory-based experiments, such as Luria–Delbrück fluctuation assays and mutation accumulation experiments, as well as being inferred from patterns of genetic polymorphism in natural populations [45,46,47,48,49]. Luria–Delbrück fluctuation assays estimate mutation rates based on selectable phenotypes arising in clonally expanding populations subjected to artificial selective pressure at the end. This method captures only mutations that confer a detectable phenotype under selection and reflect a non-neutral subset of mutations [48]. In contrast, mutation accumulation experiments aim to estimate the baseline neutral mutation rate by imposing replicate lines through repeated single-cell bottlenecks, thereby minimizing the efficacy of selection. After many generations, accumulated mutations are identified by comparing evolved genomes to their ancestral state [50]. Although mutation accumulation experiments provide a more direct estimate of spontaneous mutation rates, they are time-intensive and may still underestimate mildly deleterious mutations that are purged over the course of the experiment.

Although informative, the above laboratory-based estimates do not capture the ecological and demographic complexity of natural populations. As an alternative, mutation rates can be inferred from observed genetic variation in natural populations [49]. These estimates are sensitive to assumptions about selection, demographic history, and effective population size. Events such as population bottlenecks and expansions can strongly bias inferred rates because genetic diversity scales with effective population size. Thus, errors in estimating effective population size or variation in effective population size can further influence mutation rate inference [44]. Consequently, inferred mutation rates typically represent long-term averages and may not reflect short-term environmental or context-dependent change. As growing empirical evidence challenges the assumption that mutation rate should be treated as a constant parameter, accurately inferring natural mutation rates and understanding the factors shaping their temporal and population-level variability have become important in population genetic studies. It is increasingly recognized that mutation rates vary substantially among species, populations, and even among strains within a species [43,46,51]. Gou et al. (2019) demonstrated variation across seven strains of *S. cerevisiae* ranging from 1.1 × 10^−7^ to 5.8 × 10^−7^ [52]. Similarly, Jiang et al. (2021) reported a 10-fold range across 16 haploid, unstressed *S. cerevisiae* strains [46]. In *Cryptococcus* spp. Xu et al. (2001) observed mutation rates ranging across 21 strains from 0.41 × 10^−9^ to 3135.36 × 10^−9^, with replicates of the same strain varying from 23.52 × 10^−9^ to 272.55 × 10^−9^ [53].

Two theoretical frameworks have been proposed to explain this variation: the drift-barrier hypothesis and the model of stabilizing selection. The drift-barrier hypothesis proposes that, because most mutations are neutral or deleterious, selection acts to minimize mutation rates until further increases in fidelity are outweighed by the energetic cost of maintaining highly accurate replication and repair [43]. Under this model, the lower bound of the mutation rate is determined by the strength of genetic drift. Thus, mutation rates are primarily shaped by selection against the accumulation of deleterious mutations, whereas selection for beneficial mutations or the cost for fidelity are assumed to be inconsequential [43,44,54].

Alternatively, the model of stabilizing selection proposes that an optimal mutation rate is determined by balancing multiple selective pressures [55]. Here, the mutation rate is jointly influenced by both genotype and environment, and reflects selection acting on deleterious mutations, beneficial mutations, and the energetic costs of replication fidelity [55]. Two forms of positive selection can modify the mutation rate. First-order selection acts directly on alleles that modify the cost of fidelity through DNA replication and repair mechanisms. Second-order selection increases the mutation rate when mutator alleles hitchhike with linked beneficial mutations [3,55]. This is particularly relevant in organisms that reproduce asexually, as mutator alleles can persist without reshuffling of alleles from meiosis [56]. If selection favouring an increased mutation rate is strong, the optimal mutation rate may be maintained above the drift barrier [46,55]. Likewise, if the pressure to maintain a high mutation rate is removed and the accumulation of deleterious mutations outweighs the benefit, the mutation rate will decrease [55]. The outcome of these opposing forces is highly context-dependent, with environmental variation altering both the costs and benefits associated with the mutation rate. Under this framework, it is expected that a higher mutation rate will be maintained under fluctuating environmental conditions, selected for in the presence of stress, and inherited [55].

Empirical evidence in *S. cerevisiae* supports the model of stabilizing selection. Liu et al. (2021) accumulated mutations across replicate lines and observed substantial heritable variation in mutation rate. They found 19 lines with a significantly higher and 13 lines with a significantly lower mutation rate than the progenitor, with some lines reducing the mutation rate by 40–50% [55]. Under a strict drift-barrier hypothesis, this level of reduction is not expected, as it implies that the initial mutation rate was maintained above the drift barrier [43]. Researchers also calculated *S. cerevisiae*’s mutation rate to be more than 3000 times higher than expected under the drift barrier [55]. In this experiment, Liu et al. (2021) further identified *PSP2* as a mutator gene, with the knockout nearly halving the mutation rate [55]. Together, these results suggest that the mutation rate is genetically maintained [51].

Under a stabilizing selection framework, it is hypothesized that mutator alleles can undergo positive selection when the benefit of increased genetic variation outweighs the cost of accumulating deleterious mutations, resulting in hypermutator strains. Once the benefit of increased genetic variation no longer exists, the selection would be expected to relax and the frequency of mutator alleles decreased. Under this hypothesis, it is expected that hypermutator strains will be maintained in a fluctuating environment [46].

Hypermutator strains are often observed across laboratory experiments, where they emerge under stressful conditions [46]. For example, exposure to mildly stressful conditions such as ferulic acid and lithium chloride significantly increase the mutation rate of *S. cerevisiae* [39]. Because selection typically acts when stress is present, it is difficult to determine if elevated mutation rates reflect selection for mutator alleles, increased DNA damage from exogenous stressors, or stress-induced disruption in replication and repair fidelity. It is important that we continue to test if hypermutator strains maintain heritable increases in the mutation rate once the stressor is removed and to examine natural genomes for known mutator alleles.

**Figure 2:**
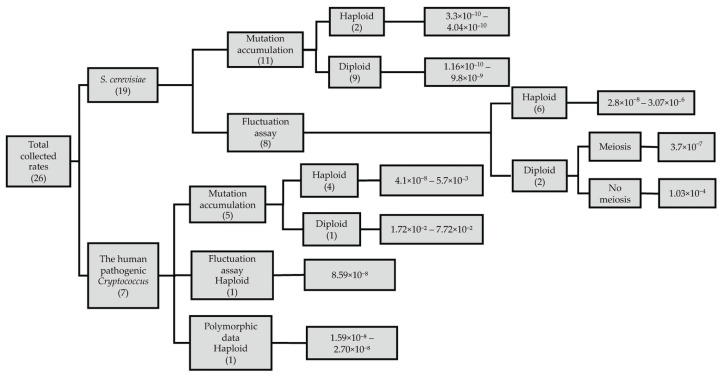
Schematic summary of reported mutations rates for *Saccharomyces cerevisiae* and the human pathogenic *Cryptococcus*. The numbers in parenthesis refer to number of reviewed publications in each category.

Hypermutators are highly relevant in human pathogenic yeasts. It is hypothesized that hypermutator phenotypes may facilitate the colonization of a host by accelerating adaptation to the harsh host environment [57,58]. Laboratory-derived mutants of HPC lacking functional *MSH2*, *MLH1*, or *PMS1* genes exhibit mutation frequencies approximately 200-fold higher than those observed in wild-type strains [57]. Clinical isolates of HPC have been identified with mutations in *MSH2* that result in significantly elevated antifungal resistance rates, exceeding 120-fold increases in certain contexts [58]. Because stress may drive selection for hypermutator strains, environmental change and antifungal exposure may elevate rates among pathogenic populations, further increasing adaptative potential. Currently, our knowledge of what drives mutator alleles in nature or how long they persist is incomplete. Jiang et al. (2021) recently identified the first natural hypermutator strain of *S. cerevisiae* associated with mosaic beer fermentation, which is noted to be a fluctuating environment [46]. The study revealed a mutation in *OGG1* that resulted in a 10-fold increased mutation rate, which was maintained under neutral laboratory conditions [46]. Additionally, by analyzing 93 clinical isolates of *S. cerevisiae*, Strope et al. (2015) identified four strains to contain an *MLH1* and *PMS1* allele combination that is engineered in laboratory strains to artificially increase the mutation rate 40-fold above the baseline rate [59,60]. However, direct mutation rate estimates obtained by Skelly et al. (2017) using fluctuation assays revealed only a 5.6-fold increase in mutation rate in these strains relative to non-mutator backgrounds, substantially lower than expected based on engineered laboratory mutators [61]. Although these clinical isolates did not exhibit mutator phenotypes in the diploid state, Raghavan et al. (2018) generated haploid spore clones from three strains and observed an approximately 340-fold range in mutation rates [62]. These results highlight the relationship between mutation and ploidy state. Table 1 and Figure 2 summarize various estimates of mutation rates for *S. cerevisiae* and HPC.

Further understanding how hypermutators emerge and persist in natural populations could strengthen our ability to predict the emergence of drug resistance and disease outbreaks, as climate change alters temperature, nutrient availability, and host–pathogen interactions in ways that may favour rapid evolution. The evolutionary consequences of mutation greatly depend on the ploidy state and rates of recombination reshuffling new variants. Thus, mutation must be considered in the context of these two processes to determine how genetic variation is generated and maintained.

**Table 1:** Summary of reported mutations rate for *Saccharomyces cerevisiae* and the human pathogenic *Cryptococcus*.

Species	Strain	Experimental Design	Ploidy	Reported Rate	Rate Calculated Based on	Meiosis	Growth Conditions	Source
*S. cerevisiae*	FY10	Mutation accumulation	Haploid	3.3 × 10^−10^	Nucleotide site per generation	No	YPD, 30 °C	Lynch et al., 2008 [63]
*S. cerevisiae*	EAY2531	Mutation accumulation	Diploid	2 × 10^−10^–3.8 × 10^−10^	Nucleotide site per generation	No	YPD, 30 °C	Nishant et al., 2010 [64]
*S. cerevisiae*	Lab strain	Mutation accumulation	Diploid	1.67 × 10^−10^	Nucleotide site per generation	No	YPD	Zhu et al., 2014 [49]
*S. cerevisiae*	SEY6211 derivatives	Mutation accumulation	Haploid	4.04 × 10^−10^	Nucleotide site per generation	No	YPD + 40 mg/L adenine sulfate, 30 °C	Sharp et al., 2018 [65]
*S. cerevisiae*	SEY6211 derivatives	Mutation accumulation	Diploid	2.89 × 10^−10^	Nucleotide site per generation	No	YPD + 40 mg/L adenine sulfate, 30 °C	Sharp et al., 2018 [65]
*S. cerevisiae*	S288C × YJM789	Mutation accumulation	Diploid	7.3 × 10^−9^–2.92 × 10^−10^	Nucleotide site per generation	No	YPD, 30 °C	Dutta et al., 2017 [66]
*S. cerevisiae*	S288C × YJM789	Mutation accumulation	Diploid	9.8 × 10^−9^	Nucleotide site per generation	No	YPD, 30 °C	Pankajam et al., 2020 [67]
*S. cerevisiae*	S288C × RM11-1a	Mutation accumulation	Diploid	1.7 × 10^−9^	Nucleotide site per generation	No	YPD, 30 °C	Pankajam et al., 2020 [67]
*S. cerevisiae*	S288C	Mutation accumulation	Diploid	1.35 × 10^−10^	Nucleotide site per generation	No	YPD, 30 °C	Pankajam et al., 2020 [67]
*S. cerevisiae*	RM11-1a	Mutation accumulation	Diploid	5.4 × 10^−9^	Nucleotide site per generation	No	YPD, 30 °C	Pankajam et al., 2020 [67]
*S. cerevisiae*	YJM789	Mutation accumulation	Diploid	1.16 × 10^−10^	Nucleotide site per generation	No	YPD, 30 °C	Pankajam et al., 2020 [67]
*S. cerevisiae*	GIL104	Fluctuation assay (*URA3 & CAN1*)	Haploid	3.07 × 10^−6^	a-Factor phenotypic resistance	No	Synthetic complete medium, 30 °C	Lang & Murray 2008 [47]
*S. cerevisiae*	GIL104	Fluctuation assay (*URA3 & CAN1*)	Haploid	1.52 × 10^−7^	10× canavanine resistance	No	Synthetic complete medium, 30 °C	Lang & Murray 2008 [47]
*S. cerevisiae*	GIL104	Fluctuation assay (*URA3 & CAN1*)	Haploid	5.43 × 10^−8^	5-FOA phenotypic resistance	No	Synthetic complete medium, 30 °C	Lang & Murray 2008 [47]
*S. cerevisiae*	Natural isolates	Fluctuation assay (*CAN1*)	NA	1.1 × 10^−7^–5.8 × 10^−7^	Canavanine phenotypic resistance	No	Synthetic complete medium, 30 °C	Gou et al., 2019 [52]
*S. cerevisiae*	YAS101, YAS106	Fluctuation assay (*CAN1*)	Haploid	9.08 × 10^−7^	Canavanine phenotypic resistance	No	YPD, 30 °C	Ohnishi et al., 2004 [68]
*S. cerevisiae*	YAS3001 (YAS101 × YAS106)	Fluctuation assay (*CAN1*)	Diploid	1.03 × 10^−4^	Canavanine phenotypic resistance	No	YPD, 30 °C	Ohnishi et al., 2004 [68]
*S. cerevisiae*	GRY2691	Fluctuation assay (*CAN1*)	Haploid	2.8 × 10^−8^	Canavanine phenotypic resistance	No	YPD, 30 °C	Rattray et al., 2015 [69]
*S. cerevisiae*	GRY3262	Fluctuation assay (*CAN1*)	Diploid	37 × 10^−8^	Canavanine phenotypic resistance	Yes	YPD, 30 °C	Rattray et al., 2015 [69]
*S. cerevisiae*	Natural isolates	Fluctuation assay (*CAN1*)	Haploid	2.1 × 10^−7^–2.1 × 10^−6^	Canavanine phenotypic resistance	No	YPD, 30 °C	Jiang et al., 2021 [46]
*Cryptococcus* spp. (*C. neoformans*)	Clinical isolates	Mutation accumulation	Haploid	0.41 × 10^−9^–3135.36 × 10^−9^	Fluconazole phenotypic resistance	No	YEPD+ fluconazole, 37 °C	Xu et al., 2001 [53]
*Cryptococcus* spp. (*C. neoformans*)	JEC50, MCC3	Mutation accumulation	Haploid	3.6 × 10^−3^–2.32 × 10^−2^	Filamentation phenotype	No	YEPD, 25 °C	Xu 2002 [70]
*Cryptococcus* spp. (*C. neoformans*)	JEC50, MCC3	Mutation accumulation	Diploid	1.72 × 10^−2^–7.72 × 10^−2^	Filamentation phenotype	Yes	YEPD, 25 °C	Xu 2002 [70]
*Cryptococcus* spp. (*C. deneoformans*)	JEC21	Mutation accumulation	Haploid	5.662 × 10^−3^	Vegetative growth	Yes	YEPD, 25 °C	Xu 2004 [71]
*Cryptococcus* spp. (*C. deneoformans*)	JEC21	Mutation accumulation	Haploid	5.332 × 10^−3^	Vegetative growth	Yes	YEPD, 37 °C	Xu 2004 [71]
*Cryptococcus* spp. (*C. deneoformans*)	JEC20a	Fluctuation assay (*FRR1*)	Haploid	8.59 × 10^−8^	Rapamycin + FK506 phenotypic resistance	No	YPD + rapamycin + FK506, 37 °C	Priest et al., 2021 [72]
*Cryptococcus* spp. (*C. gattii*)	134 natural isolates	Polymorphic data	Haploid	1.59 × 10^−8^–2.70 × 10^−8^	Nucleotide site per generation	NA	Bayesian evolutionary analysis by sampling trees (BEAST)	Roe et al., 2018 [73]

## 4. Ploidy

Populations of haploid and diploid yeast frequently exhibit distinct evolutionary dynamics in experimental systems. However, factors underlying these differences remain unclear. It is likely that differences in repair pathways, replication fidelity, and selection pressures between ploidy states contribute to this pattern, but their relative contributions remain to be fully elucidated [65,74,75].

The ploidy state across many yeast species is dynamic and often unstable, making it essential to accurately detect and characterize ploidy during evolutionary experiments. Flow cytometry is a widely used method for determining cell ploidy, as it estimates DNA content by staining nucleic acids with fluorescent dyes and quantifying fluorescence intensity at the single-cell level [76]. This approach enables quick discrimination between ploidy states and can be used to identify heterogeneity of ploidy within a population. However, measurements can be influenced by confounding factors such as cell size and cell cycle stage. In addition, flow cytometry cannot provide a chromosome-specific copy number, limiting the ability to detect aneuploidy and structural variation, but provides an initial step. Chang et al. (2024) demonstrated that exposure to fluconazole can influence flow cytometry profiles in *Cryptococcus* species and compared commonly used fluorescent dyes across *Cryptococcus* spp. and *S. cerevisiae*, highlighting the importance of a strong condition-specific control [77].

Genomic approaches allow further characterization of ploidy at the whole-genome and chromosome-specific levels. In this case, ploidy can be inferred from sequencing data by comparing observed allele frequency distributions to those expected under different ploidy states (e.g., ~0.5 in diploids, ~0.33 and ~0.66 in triploids, and ~0.25, 0.5, and ~0.75 in tetraploids) [78,79]. Read depth coverage relative to a reference genome, often examined in genomic bins, can be used to identify chromosome-specific aneuploidy and gene copy number variations [80,81]. However, genomic approaches require a strong reference genome to make inferences and high-quality sequencing data. Recently, Soraggi et al. (2022) aimed to mitigate these limitations by developing a maximum likelihood method that infers ploidy based on allelic variation and read depth, which has been shown to work with low coverage data [82]. This approach can be utilized for population genomic analyses of natural isolates, where sequencing depth and sample quality may vary.

In addition, mutation accumulation experiments (i.e., without selection) can be used to investigate ploidy dynamics over time, while an experimental evolution approach (i.e., with selection) can be used to test how different ploidy states behave under distinct selective pressures and how this impacts fitness. These methods can be used alongside genetic manipulation to identify genes involved in DNA repair pathways that maintain genome stability or contribute to polyploid formation. Failure to account for ploidy variation within a population has the potential to bias estimates of mutation rates and population genomic inferences.

Comparative studies of spontaneous mutation rate across ploidy revealed that diploids do not simply accumulate twice as many mutations as haploids, indicating that ploidy influences the mechanisms of DNA replication and repair as well as the adaptive potential of cells [65]. Mourrain et al. (2021) compared the major DNA repair pathways between haploid and diploid cells, emphasizing the distinct nature of haploid cells that lack homologous chromosomes to be used as a genetic template for repair [83]. Li et al. (2011) established that under replication stress, early events associated with ploidy determine which DNA repair pathway is activated [84]. By comparing *S. cerevisiae* haploid and diploid cells, the researchers showed that haploids engage Rad6-dependent post-replication repair pathways, where diploids rely on the Rad52- and MRX-dependent homologous recombination pathways. Li et al. (2011) established that this differential pathway usage was dictated by ploidy state, rather than mating-type locus heterozygosity or differential availability of repair enzymes [84]. Indeed, utilization of different DNA repair pathways not only influences the mutation rate but also the spectrum of mutational type.

In a study that spanned 51 strains across 33 environmental conditions, the relative fitness advantage of haploids versus diploids was found to depend strongly on the type of stressor [85]. It is hypothesized that recessive deleterious mutations would be masked in diploids. However, accumulated single nucleotide mutations demonstrated a stronger negative effect on diploids than on haploids in mutation accumulation experiments, leading to a reduced average growth rate in diploid lines. An explanation for this pattern could be that selective pressure against mutations could be more uniformly applied or stronger in haploids than in diploids [65]. Haploid *S. cerevisiae* cells accumulate approximately 40% more spontaneous single-nucleotide mutations per nucleotide site than genetically identical diploid cells, consistent with enhanced replication fidelity in diploids [65]. Sharp et al. (2018) further demonstrated that the genomic distribution of single nucleotide mutations differs significantly between haploid and diploid *S. cerevisiae* strains, suggesting that ploidy influences both the rate and the spatial pattern of mutations [65].

In contrast to point mutations, structural mutations are substantially more common in diploids than in haploids, with some experiments reporting nearly twice as many whole-chromosome changes per cell division in diploids [65]. Diploid *S. cerevisiae* cells are also expected to undergo approximately one loss-of-heterozygosity event per mitotic division, and, in heterozygous diploids, these events can rapidly generate homozygosity for advantageous alleles [86]. Similar patterns are observed in HPC, where disrupted meiosis during hybridization frequently results in diploid or aneuploid hybrids [87]. Zhu et al. (2016) identified a third of clinical *S. cerevisiae* isolates analyzed to be aneuploids (>2n) [80]. Aneuploidy in both *S. cerevisiae* and HPC contributes to substantial genome plasticity and has been repeatedly associated with adaptive responses. In HPC, aneuploidy has been shown to enhance drug resistance through gene dosage effects mediated by increased chromosome copy number [88]. By shifting ecological ranges, which bring previously isolated populations of HPC together, climate change has the potential to increase the likelihood of hybridization. Increased hybridization events can further drive genomic alterations and potentially accelerate evolutionary responses to host immune and antifungal treatments [89,90]. Understanding how ploidy and hybridization operate alongside mutation and recombination will be critical for predicting disease emergence from yeast populations.

## 5. Recombination

Recombination can occur during both mitotic and meiotic replications. During mitotic replication, recombination contributes to loss-of-heterozygosity events, while during meiosis, sexual recombination can accelerate adaptation by reshuffling genetic material. Meiotic recombination creates novel allele combinations, facilitates the purge of deleterious mutations, and increases the pool of genetic variation available for selection to act. Experimental evolution of *S. cerevisiae* has demonstrated that sexual recombination can increase the efficacy of natural selection in adapting populations. However, this advantage is strongly dependent on the environment [91]. If a clonal genotype has optimized fitness in relation to the environment, recombination is not only an energetically costly event, but it can risk decreasing the fitness level [91]. The initiation of sexual reproduction is frequently associated with stressful environmental conditions, such as nutrient depletion, high temperatures, and oxidative stress. These conditions likely favour the production of spores for long-term survival while simultaneously generating a pool of diverse genotypes with different fitness spectra across environments through recombination and assortment of genetic material [92].

In addition to reshuffling existing genetic material, meiotic recombination is inherently mutagenic, generating localized de novo genetic variation. Recombination is initiated by Spo11-mediated programmed double-strand breaks (DSBs), which are subsequently repaired. Repair of mitotic DSBs has been shown to elevate mutation rates by approximately 100–1000-fold in regions proximal to break sites, and Rattray et al. demonstrated that this mutagenic effect extends to meiotic recombination [69]. Mutation rates were correlated with local recombination rates across the genome and increased approximately 4–21-fold depending on proximity to recombination cold- and hot-spots. Disruption of Spo11 prevented the observed elevation in mutation rate, establishing the Spo11-induced DSB formation necessary for increased mutagenesis and this is further supported by an error-prone DNA repair polymerase [69]. These findings demonstrate that meiotic recombination contributes to genetic diversity by generating novel allelic combinations and by introducing de novo mutations during DSB repair. This supports the idea that recombination landscapes could be regulated, with essential genes maintained within recombination cold-spots and loci, where increased variability is advantageous when positioned near hot-spots. The regulated recombination landscape could enable the adaptive benefits of meiotic recombination while limiting the accumulation of deleterious mutations in essential genomic regions.

Genome-wide analyses demonstrate that meiotic recombination hot- and cold-spots shift with temperature, which highlights that recombination patterns can be sensitive to the environment [93].The formation of interspecies hybrids within HPC enables divergent genomes to mix, producing offspring that can display hybrid vigour by producing novel phenotypes, which enhance stress tolerance [87,94,95]. These traits can result in a fitness advantage in new or challenging environments, such as within a host or in the presence of antifungal treatments. The increasing prevalence of hybrids in clinical settings suggests that hybridization is an ongoing evolutionary force driven by the enhanced adaptive potential of hybrid genotypes [90,96,97]. For example, a whole-genome analysis of 144 *S. cerevisiae* strains identified that three clinical isolates contained genomic regions of two closely related species, *Saccharomyces paradoxus* and *Saccharomyces kudriavzevii* [80]. However, accurately estimating recombination rates in natural populations remains challenging. Recombination rates are often heterogeneous across genomes, varying among chromosomes, genomic regions, strains, life stages, and environmental conditions, which complicates efforts to define a single, representative population-level rate [98,99]. In natural populations, recombination is typically inferred indirectly from patterns of linkage disequilibrium or phylogenetic incompatibility, approaches that rely on assumptions such as the infinite sites model [100]. Violations to these assumptions and past demographic events can bias recombination signals [101]. As well, a comprehensive understanding of environmental triggers that initiate recombination is largely unresolved in most species, making it difficult to determine how frequently recombination occurs in natural populations and how it varies across environments.

Although meiotic recombination appears infrequent in natural populations of *S. cerevisiae* and HPC, genomic data provide evidence that it does occur [28,102,103,104]. Recent analyses have revealed signatures of recombination in a natural population of HPC composed almost exclusively of a single mating type (*MAT*α), indicating that sexual reproduction may occur more frequently than previously expected [105]. The most recent outbreak of cryptococcosis, which primarily impacted immunocompetent individuals, is thought to have resulted from clonal expansion of a genotype derived from sexual reproduction. It is hypothesized that recombination between two lineages produced a genotype able to expand into Vancouver Island as a new ecological niche [106,107]. These strains also demonstrated increased fertility and virulence, illustrating how meiotic recombination can drive the emergence of novel pathogenic strains [108,109,110]. Additionally, clinical isolates of *S. cerevisiae* demonstrate higher levels of heterozygosity compared to non-clinical isolates, consistent with the importance of outcrossing of the human pathogenic population of this model yeast [86].

In addition to meiotic processes, mitotic recombination, which occurs during vegetative growth of diploid or aneuploid cells, can contribute to loss of heterozygosity, gene conversion, or chromosomal rearrangements [111,112]. Under stress, these chromosomal changes can fix advantageous alleles and drop deleterious alleles in diploid cells without requiring meiosis, allowing for quick adaptation. This mechanism is often observed in the presence of antifungal and oxidative pressure [90,94,113,114]. Thus, recombination is a powerful evolutionary force generating diversity under stress and enabling rapid adaptation, yet the frequency and drivers of recombination in natural populations remain unresolved for these and other fungi, making it difficult to understand how recombination, mutation and ploidy interact to shape adaptive potential.

## 6. Future Directions

**Figure 3:**
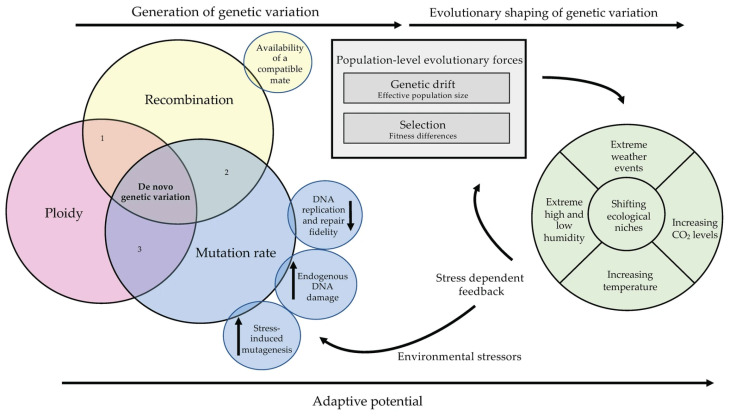
Conceptual depiction of the interconnection between ploidy, recombination and mutation to generate genetic variation to be shaped by population-level evolution forces and environmental factors.

Even though ample work has been completed to characterize the roles of mutation, ploidy and recombination in maintaining genetic variation, many unresolved questions remain. These processes are typically studied independently under controlled laboratory conditions, which fail to reflect the true complexity of natural populations. As this review highlights, these processes are interdependent and additionally influenced by life cycle, environment and population history, and the interaction of these factors determines a population’s ability to adapt. Their relationships and impacts on genetic variation and adaptation are summarized in Figure 3. Thus, it is important that future research aims to address how these factors act beyond controlled, single-parameter estimates to improve our capacity for predicting how genetic variation is maintained across changing environments in complex natural systems.

Although fundamental to our understanding, dependence on laboratory-derived parameter estimates alone restricts the extent to which current knowledge can be generalized to natural systems. Due to the nature of experimental design, mutation, ploidy and recombination are often tested under a constant environment with fixed environmental parameters. While such designs can provide a sense for how these processes work, they fail to capture the true complexity of the system. In addition, current designs often rely on single-colony transfers that impose relaxed selection through population bottlenecks and are conducted under relatively short timeframes. Even with artificially induced selection, most experiments often investigate a single adaptative trait and are restricted to clonal lineages, simplifying how adaptation is governed under natural conditions.

Considering that both *S. cerevisiae* and HPC only undergo sexual reproduction under specific conditions, which are not fully elucidated in nature, designing experiments which reflect natural life cycle variation remains difficult. If sexual reproduction does not occur within an experimental design, it is unclear if this reflects a biological constraint or if it is due to conditions which do not reflect what occurs in nature. In these species, outcrossing is expected to occur infrequently over a long period and depends on mating type compatibility, which may not be fully captured in a typical laboratory experiment. In addition, research often restricts experiments to haploid, clonal lineages with manipulated genetic backgrounds that aim to supress the likelihood of meiosis. For example, most experiments of *S. cerevisiae* are done using haploid strains, although the diploid state is considered more frequent in nature. Ploidy changes can influence both reproductive mode and genetic diversity, yet most experiments aim to limit variation in ploidy state by starting with a defined ploidy background. Ploidy shifts that occur during an experiment are often excluded and treated as experimental noise, generating a biased representation of how variation in ploidy across a population may influence genetic variation.

Additionally, most research is completed using a subset of laboratory strains that reflect a similar genetic background. These strains are often optimized for controlled laboratory conditions, which decreases the pressure for adaptative processes. Long-term culturing of these strains can lead to an accumulation of laboratory-specific traits, which do not reflect local adaptation and the demographic history of natural strains. Therefore, strains used for experimental design do not represent the true diversity and population structure of natural populations and overlook divergent subpopulations. For example, a natural *S. cerevisiae* population was found to demonstrate a highly elevated rate of C → A mutations, which distinguishes it from traditional lab strains [46]. To address this limitation, environmental sampling of yeast populations across heterogenous ecological niches is required. By doing so, researchers can identify rare genotypes and begin to capture a more realistic representation of genetic variation across natural populations. Additionally, whole-genome sequencing of natural isolates is required to elucidate cryptic variation and detect rare signals of recombination and ploidy changes. Combining whole-genome polymorphic data analysis with comprehensive sampling data and in vitro examination of natural strains will greatly improve our overall understanding of how these processes shape genetic variation.

The role of lateral gene transfer in fungal evolution should also be critically examined further. Although traditionally considered rare in eukaryotes, increasing genomic evidence suggests that horizontal gene transfer may contribute to rapid adaptation in fungal pathogens [115]. Lateral gene transfer complicates the inference of mutation, ploidy and recombination because it can lead to signals which bias the interpretation of these processes. Thus, understanding how lateral gene transfer interacts with mutation, recombination, and ploidy will be critical for fully elucidating how genetic variation is maintained across natural populations.

As climate change continues to increase the instability of environmental conditions, it is expected that altering temperature and humidity averages, shifting ecological boundaries, and increasing overlap among previously isolated populations will continue to drive the adaptation of natural yeast populations, including known and emerging human pathogenic yeasts [22,116,117,118,119,120,121]. This makes it increasingly more important to recognize how mutation, ploidy and recombination work interdependently to maintain genetic variation and drive adaptation to heterogenous environmental conditions.

## Data Availability

No new data were created or analyzed in this study. Data sharing is not applicable to this article.
